# EXpert consensus On Diaphragm UltraSonography in the critically ill (EXODUS): a Delphi consensus statement on the measurement of diaphragm ultrasound-derived parameters in a critical care setting

**DOI:** 10.1186/s13054-022-03975-5

**Published:** 2022-04-08

**Authors:** Mark E. Haaksma, Jasper M. Smit, Alain Boussuges, Alexandre Demoule, Martin Dres, Giovanni Ferrari, Paolo Formenti, Ewan C. Goligher, Leo Heunks, Endry H. T. Lim, Lidwine B. Mokkink, Eleni Soilemezi, Zhonghua Shi, Michele Umbrello, Luigi Vetrugno, Emmanuel Vivier, Lei Xu, Massimo Zambon, Pieter R. Tuinman

**Affiliations:** 1 Department of Intensive Care Medicine, Amsterdam University Medical Centers, location VUmc, Postbox 7507, 1007MB Amsterdam, The Netherlands; 2 Amsterdam Leiden Intensive Care Focused Echography (ALIFE, www.alifeofpocus.com), Amsterdam, The Netherlands; 3 Amsterdam Cardiovascular Sciences Research Institute, Amsterdam UMC, Amsterdam, The Netherlands; 4 Aix Marseille Université, Center for Cardiovascular and Nutrition Research (C2VN), INSERM, INRAE, and Service d’Explorations Fonctionnelles Respiratoires, CHU Nord, Assistance Publique Des Hôpitaux de Marseille, Marseille, France; 5 AP-HP, Groupe Hospitalier Universitaire APHP-Sorbonne Université, Site Pitié-Salpêtrière, Service de Médecine Intensive Et Réanimation (Département R3S), and Sorbonne Université, INSERM, UMRS1158 Neurophysiologie Respiratoire Expérimentale Et Clinique, 75005 Paris, France; 6 Pneumologia E Unità Di Terapia Semi Intensiva Respiratoria, AO Umberto I Mauriziano, Turin, Italy; 7 SC Anestesia E Rianimazione, Ospedale San Paolo – Polo Universitario, ASST Santi Paolo eCarlo, Milan, Italy; 8 Interdepartmental Division of Critical Care Medicine, University of Toronto, Toronto, Canada; 9 Department of Medicine, Division of Respirology, University Health Network, Toronto, Canada; 10 Toronto General Hospital Research Institute, Toronto, Canada; 11 Department of Intensive Care Medicine, Erasmsus University Medical Center, Rotterdam, The Netherlands; 12 Department of Epidemiology and Data Science, Amsterdam Public Health Research Institute, Amsterdam UMC, Vrije Universiteit Amsterdam, Amsterdam, The Netherlands; 13 Department of Intensive Care Medicine, Papageorgiou General Hospital, Thessaloniki, Greece; 14 Departement of Intensive Care Medicine, Beijing Sanbo Brain Hospital, Capital Medical University, Beijing, China; 15 SC Anestesia E Rianimazione II, Ospedale San Carlo Borromeo, ASST Santi Paolo E Carlo Polo Universitario, Milan, Italy; 16 Department of Medical, Oral and Biotechnological Sciences, University of Chieti-Pescara, Chieti, Italy; 17 Department of Anesthesiology, Critical Care Medicine and Emergency, SS. Annunziata Hospital, Chieti, Italy; 18 Médecine Intensive Réanimation, Centre Hospitalier Saint Joseph Saint Luc, Lyon, France; 19 Department of Neurosurgery and Neurosurgical Intensive Care Unit, Chongqing Emergency Medical Centre, Chongqing University Central Hospital, Chongqing, China; 20 Department of Anaesthesia and Intensive Care, Ospedale Di Cernusco Sul Naviglio, ASST Melegnano-Martesana, Milan, Italy

**Keywords:** Diaphragm, Ultrasound, Delphi, Consensus

## Abstract

**Background:**

Diaphragm ultrasonography is rapidly evolving in both critical care and research. Nevertheless, methodologically robust guidelines on its methodology and acquiring expertise do not, or only partially, exist. Therefore, we set out to provide consensus-based statements towards a universal measurement protocol for diaphragm ultrasonography and establish key areas for research.

**Methods:**

To formulate a robust expert consensus statement, between November 2020 and May 2021, a two-round, anonymous and online survey-based Delphi study among experts in the field was performed. Based on the literature review, the following domains were chosen: “Anatomy and physiology”, “Transducer Settings”, “Ventilator Impact”, “Learning and expertise”, “Daily practice” and “Future directions”. Agreement of ≥ 68% (≥ 10 panelists) was needed to reach consensus on a question.

**Results:**

Of 18 panelists invited, 14 agreed to participate in the survey. After two rounds, the survey included 117 questions of which 42 questions were designed to collect arguments and opinions and 75 questions aimed at reaching consensus. Of these, 46 (61%) consensus was reached. In both rounds, the response rate was 100%. Among others, there was agreement on measuring thickness between the pleura and peritoneum, using > 10% decrease in thickness as cut-off for atrophy and using 40 examinations as minimum training to use diaphragm ultrasonography in clinical practice. In addition, key areas for research were established.

**Conclusion:**

This expert consensus statement presents the first set of consensus-based statements on diaphragm ultrasonography methodology. They serve to ensure high-quality and homogenous measurements in daily clinical practice and in research. In addition, important gaps in current knowledge and thereby key areas for research are established.

*Trial registration* The study was pre-registered on the Open Science Framework with registration digital object identifier https://doi.org/10.17605/OSF.IO/HM8UG.

**Supplementary Information:**

The online version contains supplementary material available at 10.1186/s13054-022-03975-5.

## Introduction

Diaphragm ultrasonography is a rapidly growing field of research, with close to 3000 PubMed-listed publications over the last decade. It has been shown to be a feasible and accurate tool to assess diaphragm anatomy, respiratory physiology and, especially in ventilated critically ill patients, pathology [1–6].

The currently most well-studied methods of diaphragm ultrasonography include assessment of changes in muscular thickness over time, contractile activity (i.e. thickening fraction) and excursion during active breathing [1, 7, 8]. With these parameters, the physician can quickly obtain valuable information at the bedside with little patient burden. Important applications include mapping loss of muscle mass through repeated measurements of thickness, determining adequate ventilatory support through assessment of excessive and insufficient contractile activity, and predict the outcome of liberation attempts from mechanical ventilation and detection of patient–ventilator interaction through temporal comparison of pressure curves of the ventilator with contractile activity and excursion of the diaphragm [9–18].

While the areas of implementation are well understood, guidelines for methodology such as transducer settings, image acquisition and ventilator impact on measurements do not, or only partially, exist and are mostly derived from narrative reviews. Significant variability in diaphragm ultrasonography methodology hampers quality and comparison of studies in this field and, consequentially, implementation in daily clinical practice.

As such, we set out to perform a Delphi process across seven categories, including diaphragm anatomy, transducers settings, image acquisition technique, limitations of mechanical ventilation through passive displacement of the diaphragm, guidance for learning and obtaining expertise and application in clinical practice. The aim of this study was to provide a consensus statement towards a universal measurement protocol for diaphragm ultrasonography in research and daily practice and determine key areas for future research.

## Methods

Between November 2020 and March 2021, international experts were invited to participate in a Delphi procedure using web-based questionnaires as method for consensus development. This method was chosen as it serves to establish consensus on topics with unclear and/or conflicting evidence, while at the same time allowing exploration of fields beyond existing knowledge [19].

Panelists were invited based on their proven expertise in diaphragm ultrasonography with prior publications. This entailed at least two peer-reviewed publications with original data of which one as leading author, with either diaphragm excursion, thickness and/or thickening as the main outcome variable in an adult critical care setting. Experts from different hospitals, countries and continents were invited to minimize risk of establishing local viewpoints as consensus.

Inception of the survey consisted of several steps. First, an epidemiologist (LM) specialized in Delphi methodology was consulted for the process design. A two-round survey was selected as appropriate method to form consensus, providing sufficient rounds to reach consensus without risking dropouts due to the extent of the survey [20]. Second, a literature review was performed of recently published (systematic-) review articles listed in PubMed on diaphragm ultrasound in critical care medicine. Based on previous knowledge on the topic and information gathered from the literature, topics relevant for the survey were established by two researchers (MH and PT). These topics were then grouped within seven overarching categories, to organize and provide better overview of the framework for a measurement protocol. These included “Anatomy and physiology”, “Transducer settings”, “Technique”, “Ventilator Impact”, “Learning and Expertise”, “Daily Practice” and “Future Directions”. Third, based on these categories, five investigators (MH, EL, PT, JS and LM) of the lead research group created the questions and statements to be included in the pilot survey. Fourth, the pilot survey was then conducted within a local expert group (HV, AJ, MW, MHe) to evaluate comprehensiveness and comprehensibility of the questionnaire and adapted accordingly.

The pilot round and both study rounds contained questions based on a 5-point Likert scale, ranging from strongly disagree to strongly agree [21]. No default answers were preselected to avoid introducing bias to the experts’ responses, and every question contained a free text response if the panelists desired provision of additional comments. In addition, the questionnaire contained open questions to explore the panelist’s views and opinions across several fields. Questions were organized by seven domains (as outlined above), each on its own page with a bar indicating progress across the questionnaire to create better overview and to minimize straightlining (selecting the same response down a line of survey answers). An a priori cut-off at ≥ 68% was determined as the minimum threshold to reach consensus on an individual question and provide a statement [22–24]. This threshold was deemed appropriate to facilitate formation of consensus while allowing for disagreement and collection of arguments in case of the latter.

**Fig. 1:**
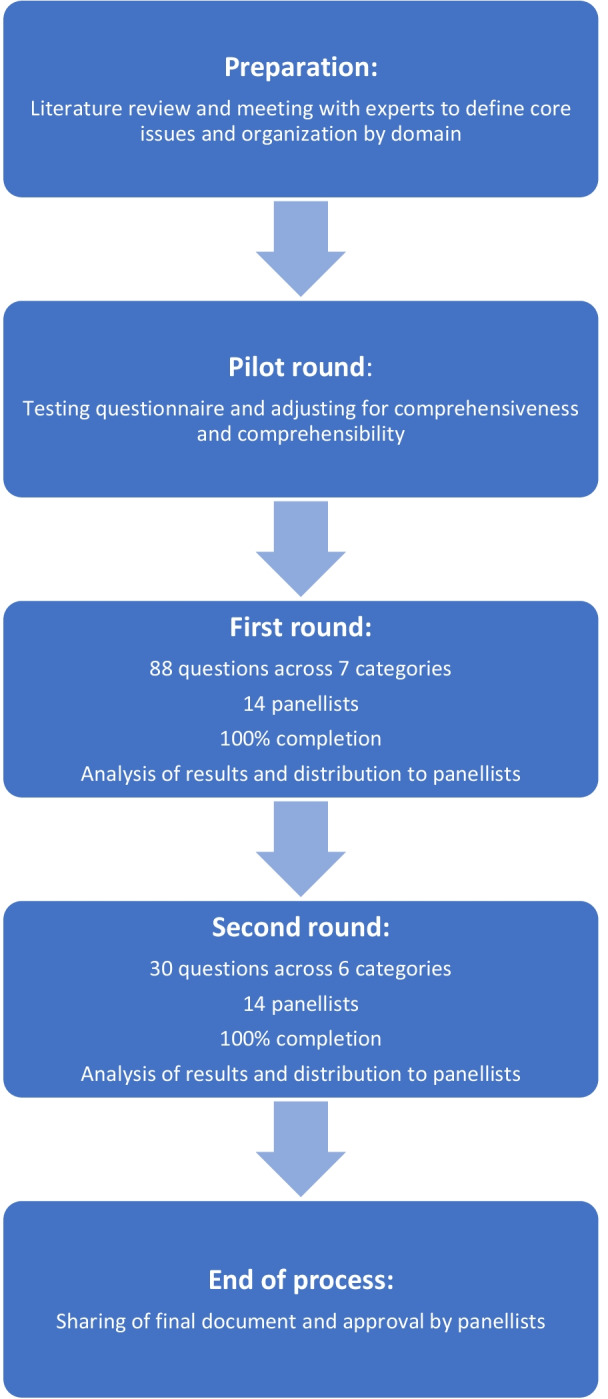
Flow chart of the study procedure

After the first round, a detailed summary of all statements and corresponding answers, arguments and percentage consensus was distributed to all panelists. All panelists were blinded to the identity of other panelists. While the steering committee was not blinded to the identity of the panelists, they were blinded to the individuals to whom the answers and arguments pertained. The second round contained modified and new questions based on the panelist’s answers and feedback from the first round. Questions on which consensus was already achieved in round one were not repeated in this round. A summary of study proceedings is provided in Fig. 1.

The study was pre-registered on the Open Science Framework with registration digital object identifier https://doi.org/10.17605/OSF.IO/HM8UG [25].

## Results

Eighteen panelists were invited to participate in the Delphi process of which 15 replied, 14 agreed to participate and 1 declined. This panel was formed by intensivists from Canada, China, France, Greece, Italy and The Netherlands. Of the 14 participants, a response rate of 100% was achieved for all questions on both rounds. A full list of experts is provided in the Acknowledgements.

In the pilot round, 89 questions were established and grouped into seven categories. Several changes were made which included omission of redundant questions, addition of new questions and changes regarding completeness and comprehensibility. This resulted in a survey with a total of 88 questions for Round 1. Of these, 35 questions were designed to collect opinions and arguments and 53 to reach consensus on the respective question. In round 1, consensus was reached on 33 questions. With answers provided from Round 1, the survey for Round 2 was established. Round 2 contained 29 new questions, of which 7 questions were designed to collect opinions and arguments and 22 to reach consensus on the respective question. In round 2, consensus was reached on 13 questions. In total, this resulted in 75 questions with the possibility for consensus across Rounds 1 and 2. Consensus was reached in 46 (61%).

A summary of the number of questions organized by rounds and categories is provided in Table 1. A more detailed overview of the questionnaire and outcome reached is provided in the Additional files 1–4. The results are summarized per category and provided in Tables 2, 3, 4 and 5. Consensus statements for anatomy and physiology are presented in Table 2. Consensus statements for transducer settings and technique are presented in Table 3. Visual examples of the statements are provided in Additional file 5. Statements for learning and expertise are presented in Table 4. Areas for future research are presented in Table 5.

**Table 1:** Summary of survey rounds

Domains	Number of questions*		Number of questions with consensus**		
	Round 1	Round 2	Round 1	Round 2	Cumulative
Anatomy and physiology	14	8	3/10	3/5	6/15
Transducer settings	15	5	7/10	5/5	12/15
Technique	15	12	2/6	3/8	5/14
Ventilator impact	10	2	7/10	1/2	8/12
Learning and expertise	11	1	2/2	1/1	3/3
Daily practice	19	1	12/15	0/1	12/16
Future directions	4	0	N.A	N.A	N.A
Total	88	29	33/53	13/22	46/75

*N.A.* not applicable (no questions aimed at reaching consensus were included)

*Questions: Includes statements and open-ended questions to gather viewpoints and arguments

**Consensus: Does not include statements and open-ended questions to gather viewpoints and arguments, Consensus was achieved with > 68% (≥ 10 respondents) agreement

**Table 2:** Diaphragm anatomy and physiology, and ventilator impact in diaphragm ultrasonography

*Anatomy and physiology*	
Anatomy Muscle No consensus was achieved on continuity of diaphragm thickness in the zone of apposition The significance of echogenicity is unknown but should be investigated Changes in thickness ≥ 10% decrease from baseline thickness is regarded as cut-off for clinically relevant atrophy No consensus was achieved regarding cut-off for increased thickness due to confounding with inflammation and oedema Limitations for measurements Obesity and large tidal volume can complicate measurements Physiology Maximum effort measurements offer important information but are hard to obtain and compare due to subjectivity of a maximum effort Dysfunction Diaphragm excursion < 2 cm is indicative of dysfunction during quiet breathing No consensus was achieved on cut-off for dysfunction based on thickening fraction	
*Ventilator impact*	
Excursion Positive pressure ventilation augments amplitude with greater lung inflation PEEP lowers diaphragm resting position and reduces excursion Thickness Positive pressure ventilation reduces patient effort and as such thickness at end inspiration PEEP lowers the diaphragm resting position with higher thickness at end expiration due to shortening of the muscle Thickening Positive pressure ventilation reduces patient effort and as such diaphragmatic thickening PEEP lowers the diaphragm resting position with higher thickness at end expiration due to shortening of the muscle and as such its percentual thickening

**Table 3:** Diaphragm ultrasonography: transducer settings and technique

*Transducer settings*	
Excursion The ideal range is between 2 and 5 MHz (cardiac or abdominal transducer) The ideal mode is the M-mode Maximum depth should be adjusted to capture maximum excursion Gain should be adjusted to create ideal contrast with surrounding structures Thickness The ideal range is between 7 and12 MHz (linear transducer) No consensus was achieved for preferring B-mode or M-mode Depth should be set just below to several centimetres under the diaphragm Gain should be adjusted to create ideal contrast with surrounding structures	
*Technique*	
Excursion The transducer should be aimed at the dome of the diaphragm No consensus was achieved on transducer placement on the abdomen Measurements are best performed in M-mode and during quiet breathing Organ displacement is a valid alternative for excursion if the diaphragm dome is hard to visualize Thickness The transducer should be placed on the midaxillary line or slightly more ventral, approximately between the 8th and 11th rib, with lung slightly or just not moving into the image The transducer should be placed perpendicular to chest wall, so that all three layers (pleura, peritoneum and fibrous layer) are visible No consensus was achieved on transducer orientation to be in line with or perpendicular to the intercostal space Caliper placement should be as close as possible to the pleural and peritoneal line without including these lines in the measurement No consensus was achieved on the optimal breathing pattern for making measurements Both Unilateral measurement of the diaphragm on the right side of the patient is an acceptable proxy for the whole diaphragm, unless there is any suspicion of unilateral pathology (e.g. thoracic surgery, phrenic nerve or spinal cord injury) in which case this needs to be excluded or measurements need to be taken on both sides

**Table 4:** Learning, expertise and applications of diaphragm ultrasonography in clinical practice

*Learning and expertise*	
Excursion Measuring diaphragm excursion is an easy skill and with steep learning curve Thickness Measuring diaphragm thickness is not an easy skill and has a slow learning curve Excursion and thickness A teaching program to learn diaphragm ultrasonography should include anatomy of the diaphragm, anatomical landmarks for measurement, supervised practice and a practical skill examination A minimum of 40 (ideally bilateral) examinations, of which at least 20 should be under (indirect) supervision of an experienced teacher, are needed for independent use in daily practice	
*Daily practice*	
Skills necessary in daily practice Excursion measurements are a necessary skill for daily practice Thickness measurements to calculate diaphragm thickening are a necessary skill for daily practice Useful indications Monitoring diaphragm function and determining dysfunction Prognostication of difficult weaning, extubation outcome and length of ICU stay Detect patient–ventilator asynchrony and titrate ventilator settings

**Table 5:** Future directives

*General* Standardization of transducer settings and technique is necessary *Basic science* Histological changes caused by ventilation should be investigated (e.g. inflammation, fibrosis, oedema) The histological basis of the middle hyperechogenic layer should be determined Cut-offs for diaphragm dysfunction in various clinical settings should be determined The interaction with other respiratory muscles, e.g. the impact of expiratory muscle atrophy/dysfunction on diaphragm function, should be investigated *Indications in clinical practice* Effective ultrasonographic parameter to accurately estimate work of breathing should be investigated The use of ultrasonography as screening tool to identify patient–ventilator asynchrony should be investigated The role of diaphragm ultrasonography to effectively titrate ventilator settings (i.e. diaphragm protective ventilation) should be investigated The role of diaphragm ultrasonography in non-invasive ventilation (e.g. as predictor of liberation from mechanical ventilation or to titrate support settings) should be investigated Automation of image acquisition *New techniques of interest for diaphragm ultrasonography* Shear wave elastography Speckle tracking Diaphragm acceleration as parameter for (dys-)function Automated image collection for monitoring purposes	

## Discussion

This Delphi study on diaphragm ultrasonography is the first consensus-based approach to formulate statements on methodology taking into account the effects of diaphragm anatomy, physiology, impact of ventilator settings on ultrasound measurements, transducer settings and technique of image acquisition. Statements for learning and reaching expertise in diaphragm ultrasonography were also formulated. Through this process, we defined areas for application in daily clinical practice, identified areas of controversy and established key opportunities for future research.

Given the rapid growth of diaphragm ultrasonography as tool in daily clinical practice and in research, a measurement protocol and recommendations for acquiring expertise in a critical care setting were urgently needed. While some clinical and literature review studies exist that address aspects of image acquisition and areas for clinical implementation, none also fully encompass the variety of additional key components such as effects of diaphragm anatomy, physiology and impact of ventilator settings on ultrasound measurements. In addition, previously reported methodologies for diaphragm ultrasonography reflected local, rather than international, consensus. With the collaboration of a large group of international experts, we aimed to overcome these limitations and generate guidelines with direct implications for clinical practice and research. In the following paragraphs, we discuss areas of consensus and controversy of special interest.

First, regarding ultrasonographic anatomy of the diaphragm, consensus was established on > 10% decrease as relevant cut-off for atrophy. This is highly relevant as it has been shown that diaphragm atrophy impacts clinical outcomes such as duration of mechanical ventilation. However, for an increase in thickness, which is equally interesting in terms of potential clinical impact, no cut-off was established. It was considered impossible to distinguish the cause for the increase in thickness, for example true muscular hypertrophy from inflammation, oedema or fibrosis. In this regard, evaluating the echogenicity of the diaphragm, and thereby potentially quality of the diaphragm, was agreed to be an area of special interest for future research [26].

Another important point of controversy was continuity of muscle thickness throughout the zone of apposition. Settling this debate is pertinent, as in case differences in thickness do exist, the location of measurement could impact the obtained thickness and derived parameters of functionality such as the thickening fraction. Available evidence is scarce and only reflects local and not global thickness [27, 28].

Second, various controversies remain regarding physiology of diaphragmatic contraction. For one, no consensus was reached on which moments of the respiratory cycle are better for taking measurements, e.g. peak inspiration versus end-inspiration. Whether these subtle differences impact final measurements is unknown and remains to be investigated. Until then, a pragmatic approach would be taking measurements of the thickest and thinnest state of the diaphragm. Another point of debate is the clinical utility of measurements of maximum effort. While panelists agreed that they theoretically provide clinically useful information about diaphragm functionality, the critical concern is variability in eliciting maximum efforts and estimating if maximum efforts were given by the patient. As follows, methods that allow standardization of eliciting maximum efforts are necessary for implementation in clinical practice [29]. An additional point of controversy was the cut-off value for diaphragm dysfunction assessed by the thickening fraction. While various cut-offs for thickening fraction as parameter for failing spontaneous breathing trials or extubation exist, the conducted studies vary strongly in terms of outcome definition and patient population [14, 18, 30, 31]. Even in healthy individuals, normal values have been shown to be highly variable and body position dependent [27, 32, 33]. We hypothesize that these aspects are key limiting factors of forming consensus, as cut-off values might vary according to the context of the measurement. As follows, determining context-specific (e.g. during (un-)assisted breathing, respiratory distress, spontaneous breathing trial, etc.) or outcome-related cut-off values (e.g. failing extubation, at risk for exhaustion and intubation, stratification of over- or under-assistance by ventilator) is an important next step.

Third, vital steps were taken towards a measurement protocol for diaphragm excursion and thickness in the critically ill. Choice of transducer, ideal depth and gain, transducer positioning and alignment in regards to the diaphragm were agreed upon. A crucial statement is that diaphragm thickness should be measured between the pleura and peritoneum, not including them into the total thickness. These are important steps in reducing heterogeneity between measurement methodology and thus increasing external validity in research and clinical practice. Nevertheless, some important aspects still remain without consensus. These include making thickness measurements in line with or crossing the intercostal space and obtaining images in B-mode or M-mode. The advantage of M-mode is allowing more accurate timing of the respiratory cycle, while advantages of B-mode are better spatial orientation and ease of use. For now, there is no evidence favouring either method and no clear advantages are directly apparent [34, 35]. Until this issue is resolved, clinicians are encouraged to use the method they are most comfortable with and researchers to clearly state their method of choice.

Fourth, essential areas of application for diaphragm ultrasonography in daily practice were determined. These included, among others, evaluating diaphragm dysfunction, prognosticating difficult weaning and detecting patient ventilator asynchrony. At the same time, factors limiting the applicability and/or interpretability of measurements were also established. In this regard, clinicians are recommended to appreciate the measurements in the light of the impact of positive end expiratory pressure on measurements, through diminished excursion and higher resting thickness due to the lower resting position [36]. The same holds true for the effect of positive pressure ventilation on reduced patient effort and passive displacement.

Lastly, a new consensus was reached on the minimum training necessary to achieve sufficient proficiency to use diaphragm ultrasound, including excursion, thickness and thickening, in clinical practice. The threshold was agreed upon to be at least 40, ideally bilaterally performed, examinations of which half should be under supervision. However, the consensus reached poses a general statement that does not take prior ultrasound experience into account [37]. In addition, we emphasize that this statement does not address the minimum number of examinations needed to obtain high reproducibility, which was demonstrated in a previous study, but the minimum training necessary for using diaphragm ultrasonography in clinical practice and guide decision-making [4].

## Strengths and limitations

There are important strengths and limitations to this study that merit consideration. First, the selection of panelists was limited to physicians with a strong scientific background. This resulted in a selected group, and local experts and educators with thorough knowledge and clinical experience but without peer reviewed publications might have been missed. Nevertheless, the advantage of this approach is guaranteed expertise with in-depth knowledge of current scientific viewpoints, which strengthens the statements formulated. Second, the classical Delphi approach does not include the possibility for a live discussion among the panelists, which could potentially help elucidate, clarify and resolve complex issues. However, it does allow for complete anonymity which prevents interference of group dynamics and provides opportunity to express unpopular or controversial opinions [38]. In addition, the possibility was presented to provide arguments for the answers selected, which were also presented to the panelists. Third, this study included two rounds. More rounds could have provided opportunity to discuss unresolved issues. Nevertheless, this did result in full completion of the surveys by all panelists, which would become less likely with increasing number of rounds [20].

## Conclusion

This expert consensus statement presents the first set of evidence-based statements on diaphragm ultrasonography methodology. They serve to ensure high-quality measurements in daily clinical practice and in research. In addition, important gaps in current knowledge and thereby key areas for future research are established.

## Supplementary Information

**Additional file 1.** Consensus round 1.

**Additional file 2.** Questions round 1.

**Additional file 3.** Consensus round 2.

**Additional file 4.** Questions round 2.

**Additional file 5.** Visual example of statements.

## Data Availability

The data sets generated and/or analysed during the current study are not publicly available to protect the privacy of participants of the Delphi study but are available from the corresponding author on reasonable request.
