# Supplementary material for: EXpert consensus On Diaphragm UltraSonography in the critically ill (EXODUS): a Delphi consensus statement on the measurement of diaphragm ultrasound-derived parameters in a critical care setting
Source: Crit Care. 2022 Apr 8;26:99. doi: 10.1186/s13054-022-03975-5 (PMC8991486; doi:10.1186/s13054-022-03975-5)
Supplement: Supplementary file 2 — Additional file 2. Questions round 1. [file 13054_2022_3975_MOESM2_ESM.docx]

EXpert Opinion On Diaphragm UltraSound

Ultrasonographic Anatomy and Physiology

Is the thickness constant throughout the entire zone of apposition when assessed by altrasound ? qq1

|  |  |  |  |  |  |  |
| --- | --- | --- | --- | --- | --- | --- |
|  | Strongly disagree | Disagree | No opinion | Agree | Strongly agree |  |
|  |  |  |  |  |  | _____ |

A hyperechoic muscle has a significant meaning: q

|  |  |  |  |  |  |  |
| --- | --- | --- | --- | --- | --- | --- |
|  | Strongly disagree | Disagree | No opinion | Agree | Strongly agree | Please specify the meaning if you (strongly) agree: |
|  |  |  |  |  |  | _____ |

Is it possible to define ultrasound cutoffs for dysfunction of the diaphragm? qq3

|  |  |  |  |  |  |  |
| --- | --- | --- | --- | --- | --- | --- |
|  | Strongly disagree | Disagree | No opinion | Agree | Strongly agree |  |
|  |  |  |  |  |  | _____ |

Based on which ultrasound parameter(s) is it possible to define dysfunction? qq4

|  |  |  |  |  |  |  |
| --- | --- | --- | --- | --- | --- | --- |
|  | On the basis of thickening | On the basis of excursion | On either thickening or excursion | Neither are feasible methods | No opinion |  |
|  |  |  |  |  |  | _____ |

What percentage of thickening would you consider as cut-off for dysfunction during normal breathing ? qq5

|  |  |  |  |  |  |
| --- | --- | --- | --- | --- | --- |
|  | 0-10% | 10-20% | 20-30% | 30-40% |  |
|  |  |  |  |  | _____ |

What percentage of thickening would you consider as cut-off for dysfunction during maximum effort ? qq6

|  |  |  |  |  |  |  |
| --- | --- | --- | --- | --- | --- | --- |
|  | 0-10% | 10-20% | 20-30% | 30-40% | 40-50% |  |
|  |  |  |  |  |  | _____ |

Which range of excursion would you consider as cutoff for dysfunction during normal breathing ? qq7

|  |  |  |  |  |  |
| --- | --- | --- | --- | --- | --- |
|  | 0-1cm | 1-2cm | 2-3cm | 3-4cm |  |
|  |  |  |  |  | _____ |

Which range of excursion would you consider as cutoff for dysfunction during maximum effort ? qq8

|  |  |  |  |  |  |  |
| --- | --- | --- | --- | --- | --- | --- |
|  | 2-3cm | 3-4cm | 4-5cm | 5-6cm | 6-7cm |  |
|  |  |  |  |  |  | _____ |

If you agree, are there any factors that could influence the above mentioned cut-offs? (multiple answers can be selected): qq9

- Sex
- Age
- Height
- Bodyweight
- Drugs
- Underlying disease (Please specific which disease(s)):_____
- Other:_____
- N/A

Is it possible to define ultrasound cut-offs for hypertrophy of the diaphragm? qq10

|  |  |  |  |  |  |  |
| --- | --- | --- | --- | --- | --- | --- |
|  | Strongly disagree | Disagree | No opinion | Agree | Strongly agree |  |
|  |  |  |  |  |  | _____ |

Is it possible to define ultrasound cut-offs for atrophy of the diaphragm? qq11

|  |  |  |  |  |  |  |
| --- | --- | --- | --- | --- | --- | --- |
|  | Strongly disagree | Disagree | No opinion | Agree | Strongly agree |  |
|  |  |  |  |  |  | _____ |

If you agree, what numerical value would you regard as a relevant cut-off for relative hypertrophy in percent and/or mm (as increases from baseline)? qq12

- 0-10%
- 10-20%
- 20-30%
- 30-40%
- 40-50%
- 0-0.5mm
- 0.5-1.0mm
- 1.0-1.5mm
- 1.5-2.0mm
- _____
- N/A

If you agree, what numerical value would you regard as relevant cut-off for atrophy in percent and/or mm (as decreases from baseline)? qq13

- 0-10%
- 10-20%
- 20-30%
- 30-40%
- 40-50%
- 0-0.5mm
- 0.5-1.0mm
- 1.0-1.5mm
- 1.5-2.0mm
- _____
- N/A

Are there patient groups that are harder to measure? qq15

- Obese
- Prone positioned
- COPD
- Large lung volume
- Irregular breathing pattern
- Increased breathing effort
- Other_____

Transducer Settings

Thickness (all questions in this section refer to thickness)

Is there an optimal transducer for thickness measurements? qa1

- Linear
- Cardiac
- Abdominal
- There is not
- No Opinion
- _____

Is there an optimal frequency for thickness measurements? qa2

- 2-5 MHz
- 5-7 MHz
- 7-12 MHz
- There is not
- No opinion
- _____

There is an optimal depth for thickness measurements: qa3

|  |  |  |  |  |  |  |
| --- | --- | --- | --- | --- | --- | --- |
|  | Strongly disagree | Disagree | No opinion | Agree | Strongly agree |  |
|  |  |  |  |  |  | _____ |

What is the optimal depth for thickness measurements? qa4

- Depth just below diaphragm
- Depth to several cm below the diaphragm
- Does not matter
- No opinion
- _____

There is an optimal gain setting for thickness measurements: qa5

|  |  |  |  |  |  |  |
| --- | --- | --- | --- | --- | --- | --- |
|  | Strongly disagree | Disagree | No opinion | Agree | Strongly agree |  |
|  |  |  |  |  |  | _____ |

How do you choose the optimal gain setting? qa6

__________

What are your personal settings of preference for thickness measurements and does your machine have presettings ? qa7

__________

Excursion (all questions in this section refer to excursion)

Is there an optimal transducer for excursion measurements? qa8

- Linear
- Cardiac
- Abdominal
- There is not
- No Opinion
- _____

Is there an optimal frequency for excursion measurements? qa9

- 2-5 MHz
- 5-7 MHz
- 7-12 MHz
- There is not
- No opinion
- _____

There is an optimal depth for excursion measurements: qa10

|  |  |  |  |  |  |  |
| --- | --- | --- | --- | --- | --- | --- |
|  | Strongly disagree | Disagree | No opinion | Agree | Strongly agree |  |
|  |  |  |  |  |  | _____ |

How do you determine this optimal depth? qa11

__________

What is the optimal depth for excursion measurements? qa12

- Depth just above the maximum distance visualized
- Depth several cm above the maximum distance visualized
- Does not matter
- No opinion
- _____

There is an optimal gain setting for excursion measurements: qa13

|  |  |  |  |  |  |  |
| --- | --- | --- | --- | --- | --- | --- |
|  | Strongly disagree | Disagree | No opinion | Agree | Strongly agree |  |
|  |  |  |  |  |  | _____ |

The optimal gain setting is: qa14

__________

What are your personal settings of preference for excursion measurements: qa15

__________

Technique

Thickness (all questions in this section refer to thickness)

In which mode are thickness measurements best performed? qc1

- In M-Mode
- In B-Mode
- It does not matter
- No opinion
- Please elucidate advantages of proposed Mode:___________

Where should the transducer be placed? (multiple answers can be selected per subject) qc2

- On midaxillary line
- More ventral from midaxillary line
- More dorsal from midaxillary line
- (Approx.) Between the 6th and 8th rib
- (Approx.) Between the 8th and 10th rib
- (Approx.) Between the 10th and 12th rib
- I do not count ribs
- Without lung moving into/out of the picture
- With lung moving into/out of the picture
- Just below lungsliding
- _____

How should the transducer be positioned in regard to the chest wall ? (multiple answers can be selected) qc3

- Perpendicular
- So that the three layers are visible
- So that the pleural line runs parallel to the ultrasound beam
- Does not matter
- No opinion
- _____

How should the transducer be positioned in regard to intercostal space? qc4

- In line with it
- Perpendicular to it
- Does not matter
- No opinion
- _____

Where should the calipers be placed when measuring thickness in relation to the pleural and peritoneal line? qc5

- On their inside, c.q. between the pleural/peritoneal lines
- On the outside, c.q. including the pleural/peritoneal lines
- It does not matter
- No opinion
- Please elucidate your answer:_____

Taking measurements on both sides of the patient is always necessary: qc6

|  |  |  |  |  |  |  |
| --- | --- | --- | --- | --- | --- | --- |
|  | Strongly disagree | Disagree | No opinion | Agree | Strongly agree |  |
|  |  |  |  |  |  | _____ |

What are situations in which both sides should be assessed? qc7

__________

When should “relaxed state” thickness be measured? qc8

- Just after expiration
- Just before inspiration
- When the diaphragm appears the thinnest
- Does not matter
- No opinion
- _____

When should “contracted state” thickness be measured? qc9

- End inspiration
- Peak inspiration
- When the diaphragm appears the thickest
- Does not matter
- No opinion
- _____

Is there a breathing pattern that is most suitable for making thickening measurements? qc10

(multiple answers possible)

- No, there is not
- Yes, during sniffing
- Yes, during quiet breathing
- Yes, during deep breathing
- No opinion
- _____

Excursion (all questions in this section refer to excursion)

In which mode are excursion measurements best performed? qc11

- In M-Mode
- In B-Mode
- It does not matter
- No opinion
- Please elucidate your answer:_____

Where should the transducer be positioned? qc12

(multiple answers can be selected)

- Midclavicular line
- Between midaxillary and midclavicular line
- Between midaxillary and sternum
- Aimed at the dome of the diaphragm (the beam reaches the dome perpendicularly)
- Aimed at the farthest point from the transducer
- Other part of the diaphragm (please specify):_____
- No opinion

How should the transducer be positioned? qc13

- Flat
- Aimed mildly dorsally
- Aimed strongly dorsally
- Aimed at the dome
- Does not matter
- No opinion
- _____

The agreement between diaphragmatic and subdiaphragmatic organ displacement (e.g. movement of liver, spleen, etc.) is high enough to use either if necessary: qc14

|  |  |  |  |  |  |  |
| --- | --- | --- | --- | --- | --- | --- |
|  | Strongly disagree | Disagree | No opinion | Agree | Strongly agree |  |
|  |  |  |  |  |  | _____ |

Is there a breathing pattern that is most suitable for making  excursion measurements? qc15

(multiple answers possible)

- No, there is not
- Yes, during sniffing
- Yes, during quiet breathing
- Yes, during deep breathing
- No opinion
- _____

Ventilator Impact

Thickness

Does pressure support ventilation impact diaphragm thickness end expiration? qb1

|  |  |  |  |  |  |  |
| --- | --- | --- | --- | --- | --- | --- |
|  | Strongly disagree | Disagree | No opinion | Agree | Strongly agree | Please specify |
|  |  |  |  |  |  | _____ |

Does pressure support ventilation impact diaphragm thickness end inspiration ? qb2

|  |  |  |  |  |  |  |
| --- | --- | --- | --- | --- | --- | --- |
|  | Strongly disagree | Disagree | No opinion | Agree | Strongly agree | Please specify |
|  |  |  |  |  |  | _____ |

Does controlled ventilation impact thickness of the diaphragm? qb3

|  |  |  |  |  |  |  |
| --- | --- | --- | --- | --- | --- | --- |
|  | Strongly disagree | Disagree | No opinion | Agree | Strongly agree | Please specify |
|  |  |  |  |  |  | _____ |

Does PEEP influence impact thickness of the diaphragm? qb4

|  |  |  |  |  |  |  |
| --- | --- | --- | --- | --- | --- | --- |
|  | Strongly disagree | Disagree | No opinion | Agree | Strongly agree | Please specify |
|  |  |  |  |  |  | _____ |

Thickening

Does pressure support ventilation impact thickening of the diaphragm? qb5

|  |  |  |  |  |  |  |
| --- | --- | --- | --- | --- | --- | --- |
|  | Strongly disagree | Disagree | No opinion | Agree | Strongly agree | Please specify |
|  |  |  |  |  |  | _____ |

Does controlled ventilation impact thickening of the diaphragm? qb6

|  |  |  |  |  |  |  |
| --- | --- | --- | --- | --- | --- | --- |
|  | Strongly disagree | Disagree | No opinion | Agree | Strongly agree | Please specify |
|  |  |  |  |  |  | _____ |

Does PEEP impact thickening of the diaphragm? qb7

|  |  |  |  |  |  |  |
| --- | --- | --- | --- | --- | --- | --- |
|  | Strongly disagree | Disagree | No opinion | Agree | Strongly agree | Please specify |
|  |  |  |  |  |  | _____ |

Excursion

Does pressure support ventilation impact excursion of the diaphragm? qb8

|  |  |  |  |  |  |  |
| --- | --- | --- | --- | --- | --- | --- |
|  | Strongly disagree | Disagree | No opinion | Agree | Strongly agree | Please specify |
|  |  |  |  |  |  | _____ |

Does controlled ventilation impact excursion of the diaphragm? qb9

|  |  |  |  |  |  |  |
| --- | --- | --- | --- | --- | --- | --- |
|  | Strongly disagree | Disagree | No opinion | Agree | Strongly agree | Please specify |
|  |  |  |  |  |  | _____ |

Does PEEP impact excursion of the diaphragm? qb10

|  |  |  |  |  |  |  |
| --- | --- | --- | --- | --- | --- | --- |
|  | Strongly disagree | Disagree | No opinion | Agree | Strongly agree | Please specify |
|  |  |  |  |  |  | _____ |

Learning and Expertise

Thickness (all questions in this section refer to thickness)

Thickness imaging of the diaphragm is an easy skill and has a steep learning curve: q55

|  |  |  |  |  |  |  |
| --- | --- | --- | --- | --- | --- | --- |
|  | Strongly disagree | Disagree | No opinion | Agree | Strongly agree |  |
|  |  |  |  |  |  | _____ |

How many patients need to be examined until general expertise is reached to make reproducible thickness images? q56

- <20
- 20-40
- 40-60
- 60-80
- >80, please specify number estimated_____
- No opinion
- _____

How many of these should be supervised? q57

- 0-10
- 10-20
- 20-30
- 30-40
- >40, please specify number estimated_____
- No opinion
- _____

How many patients need to be examined until thickness can be used to guide clinical practice? q58

- <20
- 20-40
- 40-60
- 60-80
- >80, please specify number estimated_____
- No opinion
- _____

How many of these should be supervised? q59

- 0-10
- 10-20
- 20-30
- 30-40
- >40, please specify number estimated_____
- No opinion
- _____

Excursion (all questions in this section refer to excursion)

Excursion imaging of the diaphragm is an easy skill and has a steep learning curve: q60

|  |  |  |  |  |  |  |
| --- | --- | --- | --- | --- | --- | --- |
|  | Strongly disagree | Disagree | No opinion | Agree | Strongly agree |  |
|  |  |  |  |  |  | _____ |

How many patients need to be examined until general expertise is reached to make reproducible excursion images? q61

- <20
- 20-40
- 40-60
- 60-80
- >80, please specify number estimated_____
- No opinion

How many of these should be supervised? q62

- 0-10
- 10-20
- 20-30
- 30-40
- >40, please specify number estimated_____
- No opinion
- _____

How many patients need to be examined until excursion can be used to guide clinical practice? q63

- <20
- 20-40
- 40-60
- 60-80
- >80, please specify number estimated_____
- No opinion
- _____

How many of these should be supervised? q64

- 0-10
- 10-20
- 20-30
- 30-40
- >40, please specify number estimated_____
- No opinion
- _____

Training program:

What should a teaching program for competency/expertise include? q65

(multiple answers are possible)

- Anatomy of the diaphragm
- Anatomical landmarks for measurement
- Thickness measurements on the right side
- Thickness measurements on the left side
- Thickness measurements on both sides
- Excursion measurements on the right side
- Excursion measurements on the left side
- Excursion measurements on both sides
- Setting in regard to form of ventilation
- Supervised practice
- Unsupervised practice
- E-learning
- Lectures
- Hands on lectures
- Theoretical knowledge exam
- Practical skill exam

Daily practice

General

Is diaphragm excursion a valuable skill for daily practice? q66

|  |  |  |  |  |  |  |
| --- | --- | --- | --- | --- | --- | --- |
|  | Strongly disagree | Disagree | No opinion | Agree | Strongly agree |  |
|  |  |  |  |  |  | _____ |

In which patients, setting and to which end is it valuable? q67

__________

Is the ability to perform excursion measurements a basic ultrasonographic skill that should be required for any intensivist? q86

|  |  |  |  |  |  |  |
| --- | --- | --- | --- | --- | --- | --- |
|  | Strongly disagree | Disagree | No opinion | Agree | Strongly agree |  |
|  |  |  |  |  |  | _____ |

Is diaphragm thickness a valuable skill for daily practice? q68

|  |  |  |  |  |  |  |
| --- | --- | --- | --- | --- | --- | --- |
|  | Strongly disagree | Disagree | No opinion | Agree | Strongly agree |  |
|  |  |  |  |  |  | _____ |

In which patients, setting and to which end is it valuable? q69

__________

Is the ability to perform thickness measurements a basic ultrasonographic skill that should be required for any intensivist? q87

|  |  |  |  |  |  |  |
| --- | --- | --- | --- | --- | --- | --- |
|  | Strongly disagree | Disagree | No opinion | Agree | Strongly agree |  |
|  |  |  |  |  |  | _____ |

Is diaphragm thickening fraction a valuable skill for daily practice? q70

|  |  |  |  |  |  |  |
| --- | --- | --- | --- | --- | --- | --- |
|  | Strongly disagree | Disagree | No opinion | Agree | Strongly agree |  |
|  |  |  |  |  |  | _____ |

In which patients, setting and to which end is it valuable? q71

__________

Is the ability to perform thickening measurements a basic ultrasonographic skill that should be required for any intensivist? q88

|  |  |  |  |  |  |  |
| --- | --- | --- | --- | --- | --- | --- |
|  | Strongly disagree | Disagree | No opinion | Agree | Strongly agree |  |
|  |  |  |  |  |  | _____ |

What is more valuable for daily practice, excursion, thickness or thickening? q72

- Excursion
- Thickness
- Thickening
- All equally important
- No opinion

Issue specific

How useful is diaphragm ultrasound to monitor diaphragm function? q73

|  |  |  |  |  |  |  |
| --- | --- | --- | --- | --- | --- | --- |
|  | Not useful at all | Not very useful | No opinion | A little useful | Very useful |  |
|  |  |  |  |  |  | _____ |

How useful is diaphragm ultrasound to monitor respiratory muscle training? q74

|  |  |  |  |  |  |  |
| --- | --- | --- | --- | --- | --- | --- |
|  | Not useful at all | Not very useful | No opinion | A little useful | Very useful |  |
|  |  |  |  |  |  | _____ |

How useful is diaphragm ultrasound to determine diaphragm dysfunction? q75

|  |  |  |  |  |  |  |
| --- | --- | --- | --- | --- | --- | --- |
|  | Not useful at all | Not very useful | No opinion | A little useful | Very useful |  |
|  |  |  |  |  |  | _____ |

How useful is diaphragm ultrasound to prognosticate extubation outcome? q76

|  |  |  |  |  |  |  |
| --- | --- | --- | --- | --- | --- | --- |
|  | Not useful at all | Not very useful | No opinion | A little useful | Very useful |  |
|  |  |  |  |  |  | _____ |

How useful is diaphragm ultrasound to prognosticate difficult weaning? q77

|  |  |  |  |  |  |  |
| --- | --- | --- | --- | --- | --- | --- |
|  | Not useful at all | Not very useful | No opinion | A little useful | Very useful |  |
|  |  |  |  |  |  | _____ |

How useful is diaphragm ultrasound to prognosticate length of ICU stay? q78

|  |  |  |  |  |  |  |
| --- | --- | --- | --- | --- | --- | --- |
|  | Not useful at all | Not very useful | No opinion | A little useful | Very useful |  |
|  |  |  |  |  |  | _____ |

How useful is diaphragm ultrasound to prognosticate mortality in ICU patients? q79

|  |  |  |  |  |  |  |
| --- | --- | --- | --- | --- | --- | --- |
|  | Not useful at all | Not very useful | No opinion | A little useful | Very useful |  |
|  |  |  |  |  |  | _____ |

How useful is diaphragm ultrasound to titrate ventilator settings? q80

|  |  |  |  |  |  |  |
| --- | --- | --- | --- | --- | --- | --- |
|  | Not useful at all | Not very useful | No opinion | A little useful | Very useful |  |
|  |  |  |  |  |  | _____ |

How useful is diaphragm ultrasound to detect patient ventilator asynchrony? q81

|  |  |  |  |  |  |  |
| --- | --- | --- | --- | --- | --- | --- |
|  | Not useful at all | Not very useful | No opinion | A little useful | Very useful |  |
|  |  |  |  |  |  | _____ |

Future directions

What are the gaps in our current knowledge that need further investigation? q82

__________

Which domain should research focus on in the upcoming year(s)? q83

__________

Are there potential applications for diaphragm ultrasound that are not yet commonly known? q84

__________

Are there other/new ultrasound measurements or techniques (aside from excursion, thickness, thickening) that might be of interest to evaluate? q85

__________

Thank you for your time, we highly appreciate your input! Your answers have been saved.
