# Supplementary material for: EXpert consensus On Diaphragm UltraSonography in the critically ill (EXODUS): a Delphi consensus statement on the measurement of diaphragm ultrasound-derived parameters in a critical care setting
Source: Crit Care. 2022 Apr 8;26:99. doi: 10.1186/s13054-022-03975-5 (PMC8991486; doi:10.1186/s13054-022-03975-5)
Supplement: Supplementary file 4 — Additional file 4. Questions round 2. [file 13054_2022_3975_MOESM4_ESM.docx]

EXpert Opinion on Diaphragm UltraSound - Round 2

Explanation

I have read and understood the format of the second Delphi round.  q

In round 2 of this Delphi process, we will revisit some previous questions based on the answers given and propose alternate statements as well. To this end, the new question will be highlighted in bold letters at the top of each question, while below a brief summary of Round 1 will be provided. Therefore, we would like to ask you to carefully read these summaries before answering the questions.

In case no explanation is provided, this is a new question that resulted from input of the panelists.

- Strongly disagree
- Disagree
- No opinion
- Agree
- Strongly agree

Ultrasonographic Anatomy and Physiology

On what do you base your opinion about the thickness throughout the zone of apposition? (e.g. experience, literature, etc. (please provide references if possible) q1

Question Round 1: Is the thickness constant throughout the entire zone of apposition when assessed by ultrasound?

Answers Round 1: In round 1, 58% of the panelists (strongly-)disagreed, 7% had no opinion and 36% agreed.

Based on this result, we wish to ask two additional questions to further elucidate this point.

__________

In our experience, differences in thickness throughout the zone of apposition are negligible. Assuming differences within a patient are present, do you think that they are large enough to be of clinical significance? (if you disagree and believe no differences are present please answer by (strongly)-disagree) q2

Question Round 1: Is the thickness constant throughout the entire zone of apposition when assessed by ultrasound?

Answers Round 1: In round 1, 58% of the panelists (strongly-)disagreed, 7% had no opinion and 36% agreed.

Based on this result, we wish to ask two additional questions to further elucidate this point.

|  | Strongly disagree | Disagree | No opinion | Agree | Strongly agree |
| --- | --- | --- | --- | --- | --- |
|  |  |  |  |  |  |

Do you think echogenicity of the muscle could potentially be a clinically relevant question (e.g. to assess fibrosis, oedema, etc) and merit further research? q3

Question Round 1: A hyperechoic muscle has a significant meaning ?

Answers Round 1: In round 1, 21% of panelists (strongly)disagreed, 29% had no opinion and 50% (strongly)-agreed.

To our knowledge, there is not enough evidence to support either claim. Therefore, we want to propose a different question

|  | Strongly disagree | Disagree | No opinion | Agree | Strongly agree |
| --- | --- | --- | --- | --- | --- |
|  |  |  |  |  |  |

A thickening fraction of less than 30% can be considered indicative of dysfunction during quiet breathing. Do you agree with this statement? q4

Question Round 1: What percentage of thickening would you consider as cut-off for dysfunction during normal breathing?

Answers Round 1: 46% of the panelists answered 10-20% thickening fraction, 46% answered 20-30% thickening fraction and 8% had no opinion.

In addition, we received some comments in regards to “normal” breathing, by which we intended to say quiet breathing (as opposed to sniffing or deep breathing for example). Based on the result and the comments we altered question and would like to propose a new statement.

|  | Strongly disagree | Disagree | No opinion | Agree | Strongly agree |
| --- | --- | --- | --- | --- | --- |
|  |  |  |  |  |  |

Why or why don’t you think that maximum effort measurements are clinically useful parameters. Please provide a brief (2-3 sentences) explanation and literature reference(s) if possible. q5

Question Round 1: What percentage of thickening would you consider as cut-off for dysfunction during maximum effort ?
Answer Round 1: 14% Of panelists selected a thickening fraction of 10-20%, 29% selected a thickening fraction of 20-30%, 29% selected 30-40% and 29% selected 40-50%

Question Round 1: Which range of excursion would you consider as cut-off for dysfunction during maximum effort ?
Answers Round 1: 27% of panelists selected an excursion of 2-3cm, 36% 3-4cm, 18% 4-5cm, 9% 5-6cm and 9% 6-7cm.

In addition, in both cases arguments were provided that these maximum effort measurements are not reliable (e.g. due to varying abdominal and thoracic compliance) or are not informative as a dysfunctional muscle cannot deliver a maximum effort

__________

A diaphragm excursion of  less than 2cm can be considered indicative of dysfunction during quiet breathing. Do you agree with this statement? q6

Question Round 1: Which range of excursion would you consider as cut-off for dysfunction during normal breathing?

Answers Round 1: 86% of respondents selected answers with below or equal to 2cm.

We therefore propose a different statement.

|  | Strongly disagree | Disagree | No opinion | Agree | Strongly agree |
| --- | --- | --- | --- | --- | --- |
|  |  |  |  |  |  |

What are your arguments why defining a cut-off for hypertrophy is or is not possible. Please provide a brief explanation (2-3 sentences) of your opinion and literature reference(s) if possible. q7

Question Round 1: Is it possible to define ultrasound cut-offs for hypertrophy of the diaphragm?

Answers Round 1: 54% of respondents (strongly-)agreed that it would be possible to do so, 31% (strongly-)disagreed and 15% had no opinion.

An argument provided for disagreeing with this question was that hypertrophy is the wrong word in this regard, as other factors can lead to an acute increase in thickness (we interpret this as oedema due to inflammation for example).

__________

>10% decrease from baseline thickness can be considered as cut-off for clinically relevant atrophy. Do you agree with this statement? q8

Question Round 1: If you agree, what numerical value would you regard as a relevant cut-off for relative atrophy in percent and/or mm (as increases from baseline)?

Answers Round 1: Upon review of the answers, we realized that we did not provide a clear format for the question (multiple choice instead of single choice). Nevertheless, 61% of respondents selected a percentage of 10% or higher and 30% 0.5-1.5mm.

Based on these answers, we provide a new statement.

|  | Strongly disagree | Disagree | No opinion | Agree | Strongly agree |
| --- | --- | --- | --- | --- | --- |
|  |  |  |  |  |  |

Transducer Settings

Thickness

The “Ideal” gain setting varies per hospital, machine and image obtained and there is no incorrect choice per se when assessing thickness as long as the diaphragm can be clearly delineated. Do you agree with this statement? q9

Question Round 1: How do you choose the optimal gain setting?
Answers Round 1: Arguments provided were (summarized and paraphrased): to get ideal/more contrast between the diaphragm and adjacent structures, depending on the view and machine and according to standardized presetting.

From these answers provided for this question, we propose two statements.

|  | Strongly disagree | Disagree | No opinion | Agree | Strongly agree |
| --- | --- | --- | --- | --- | --- |
|  |  |  |  |  |  |

Gain should be used to create “ideal” contrast to clearly delineate the diaphragm from surrounding structures when assessing thickness. Do you agree with this statement? q10

Question Round 1: How do you choose the optimal gain setting?
Answers Round 1: Arguments provided were (summarized and paraphrased): to get ideal/more contrast between the diaphragm and adjacent structures, depending on the view and machine and according to standardized presetting.

From these answers provided for this question, we propose three statements.

|  | Strongly disagree | Disagree | No opinion | Agree | Strongly agree |
| --- | --- | --- | --- | --- | --- |
|  |  |  |  |  |  |

Excursion

Both the cardiac and abdominal transducer are suitable for excursion measurements.
Do you agree with this statement? q12

Question Round 1: Is there an optimal transducer for excursion measurements?
Answers Round 1: The answers were equally distributed among the cardiac and abdominal transducer (50% and 50%). We therefore propose a different statement.

|  | Strongly disagree | Disagree | No opinion | Agree | Strongly agree |
| --- | --- | --- | --- | --- | --- |
|  |  |  |  |  |  |

The ideal depth for excursion measurements is patient dependent and should be chosen so that the maximum depth captures the maximal excursion of the diaphragm. Do you agree with this statement? q13

Questions Round 1: What is the optimal depth for excursion measurements?
How do you determine this optimal depth?

Answers Round 1: These arguments were (summarized and paraphrased): depending on the patient and their physique, just above the diaphragm, sufficient depth to visualize the entire respiratory cycle.

Based on these arguments, we propose a different statement.

|  | Strongly disagree | Disagree | No opinion | Agree | Strongly agree |
| --- | --- | --- | --- | --- | --- |
|  |  |  |  |  |  |

The optimal gain setting is image dependent and should be chosen so that the diaphragm is clearly visualized and discernible from adjacent structures, but dependent on the image obtained. Do you agree with this statement? q14

Questions Round 1: There is an optimal gain setting for excursion measurements: // The optimal gain setting is:

Answers Round 1: 61% of respondents (strongly-)agreed that there is an optimal gain setting while 31% were neutral and 8% (strongly-)disagreed in this regard. Arguments provided were (summarized and paraphrased): depending on the image to get the highest contrast and a dark image with little to no gain.

From these answers, we provide a new statement.

|  | Strongly disagree | Disagree | No opinion | Agree | Strongly agree |
| --- | --- | --- | --- | --- | --- |
|  |  |  |  |  |  |

Technique

Thickness

In which mode are thickness measurements best performed? q15

This question was provided in an incorrect format (multiple choice instead of single choice), for which reason multiple answers were selected. We apologize for this inconvenience and pose the same question again.

|  | B-mode | No opinion | Both B- and M-mode are equally good | M-Mode |
| --- | --- | --- | --- | --- |
|  |  |  |  |  |

Please provide a brief explanation of the answer given, potentially with reference(s) if possible. q16

__________

Both B-mode and M-mode are acceptable alternatives if the ultrasonographer has a strong preference for one. Do you agree with this statement? q17

|  | Strongly disagree | Disagree | No opinion | Agree | Strongly agree |
| --- | --- | --- | --- | --- | --- |
|  |  |  |  |  |  |

The transducer should be placed on the midaxillary line or slightly more ventral, approximately between the 8th and 11th rib, with lung slightly or just not moving into the image. Do you agree with this statement? q18

Question Round 1: Where should the transducer be placed for thickness measurements ?
Answers Round 1: A lot of insightful answers were gained from the answers provided. A complete summary of these is provided in the supplementary material. The most frequently highlighted points were summarized and rephrased into the statement above.

|  | Strongly disagree | Disagree | No opinion | Agree | Strongly agree |
| --- | --- | --- | --- | --- | --- |
|  |  |  |  |  |  |

To assess diaphragm muscle thickness, caliper placement should be done so that only muscle thickness is measured, i.e. as close as possible to the pleural and peritoneal line without including these lines in the measurement. Do you agree with this statement? q19

Question Round 1: Where should the calipers be placed when measuring thickness in relation to the pleural and peritoneal line?

Answers Round 1: 21% of respondents stated that the pleural/peritoneal lines should be included, 57% that the measurement should be performed inside these lines, 14% answered with a slight variation on this with caliper placement on the interface of- or on these lines and 7% answered that it does not matter.

No arguments were provided in favour of including the lines while arguments against doing so were (summarized and rephrased): The lines have their own thickness and should therefore not be included, the lines are not anatomical structures identified by ultrasound per se but should be regarded as artefacts and therefore be excluded from the measurement. Others argued that it does not matter as long as measurements are done consistently.

In our opinion consensus is desirable in this matter. Therefore, we would like you to reconsider this question based on the answers and arguments provided.

|  | Strongly disagree | Disagree | No opinion | Agree | Strongly agree |
| --- | --- | --- | --- | --- | --- |
|  |  |  |  |  |  |

If you do not agree with the previous statement, could you please provide arguments for your choice (2-3 sentences) and literature reference if possible. q20

__________

Unilateral measurement of the diaphragm on the right side of the patient is an acceptable proxy for the whole diaphragm, unless there is any suspicion of unilateral pathology (e.g. thoracic surgery, phrenic nerve or spinal cord injury) in which case this needs to be excluded or measurements need to be taken on both sides. Do you agree with this statement? q21

Questions Round 1: Taking measurements on both sides of the patient is always necessary? and What are situations in which both sides should be assessed?

Answers Round 1: 50% of respondents answered that unilateral measurement is sufficient while 50% stated that bilateral measurement is necessary.

A lot of arguments were provided, which are (summarized and paraphrased): Always in every patient as atrophy or dysfunction can be asymmetric or only upon suspicion of unilateral disease such as phrenic nerve injury after cardiac surgery, abdominal surgery close to the diaphragm or pleural effusion.

Based on the arguments provided, we would like to follow up this question with a new statement.

|  | Strongly disagree | Disagree | No opinion | Agree | Strongly agree |
| --- | --- | --- | --- | --- | --- |
|  |  |  |  |  |  |

Do you think that differences in measuring RELAXED state as stated in the previous round (just after expiration, just before inspiration, when the diaphragm appears thinnest) impact the obtained measurements in a clinically relevant matter? q22

Question Round 1: When should “relaxed state” thickness be measured?
Answers Round 1: 36% of respondents stated “just after expiration”, 43%” just before inspiration” and 21% when the diaphragm appears thinnest.

Based on the answers provided, we would like to follow up these questions with two other questions.

|  | Strongly disagree | Disagree | No opinion | Agree | Strongly agree |
| --- | --- | --- | --- | --- | --- |
|  |  |  |  |  |  |

Based on which physiological process do you think this matters? Please provide your opinion/and or literature reference(s). q23

Question Round 1: When should “relaxed state” thickness be measured?
Answers Round 1: 36% of respondents stated “just after expiration”, 43%” just before inspiration” and 21% when the diaphragm appears thinnest.

Based on the answers provided, we would like to follow up these questions with two other questions.

__________

Do you think that differences in measuring CONTRACTED state as stated in the previous round (end inspiration, peak inspiration, when the diaphragm appears thickest) impact the obtained measurements in a clinically relevant matter? q24

Question Round 1: When should “contracted state” thickness be measured?
Answers Round 1: 21% of respondents stated end inspiration, 57% peak inspiration and 21% when the diaphragm appears thickest.

Based on the answers provided, we would like to follow up these questions with two other questions.

|  | Strongly disagree | Disagree | No opinion | Agree | Strongly agree |
| --- | --- | --- | --- | --- | --- |
|  |  |  |  |  |  |

 Based on which physiological process do you think this matters? Please provide your opinion/and or literature reference(s). q25

Question Round 1: When should “contracted state” thickness be measured?
Answers Round 1: 21% of respondents stated end inspiration, 57% peak inspiration and 21% when the diaphragm appears thickest.

Based on the answers provided, we would like to follow up these questions with two other questions.

__________

Quiet breathing is the most suitable breathing pattern for making thickening measurements when assessing diaphragm function. Do you agree with this statement? q26

Question round 1: Is there a breathing pattern that is most suitable for making thickening measurements?
Answers round 1: This was a multiple-choice question, where the most frequently chosen answer (9 times) was that quiet breathing as the most suitable breathing pattern to assess thickening. This was followed by deep breathing (5 times), sniffing (4 times) and no particular pattern (2 times).

Some arguments were provided, that this is dependent on the aim of the measurements, e.g. to measure effort or function.

Taking this into account, we rephrase adjusted the question and propose a new statement.

|  | Strongly disagree | Disagree | No opinion | Agree | Strongly agree |
| --- | --- | --- | --- | --- | --- |
|  |  |  |  |  |  |

Ventilator impact

Thickness

Does pressure support ventilation impact diaphragm thickness end expiration? q27

Question Round 1: Does pressure support ventilation impact diaphragm thickness end expiration? 
Answers Round 1: 50% of the respondents answered (strongly)-disagree) while 35%(strongly-) agreed and 14% had no opinion.

Reasons provided for agreeing with this statement were that PEEP is the culprit in this regard. We want to highlight that PEEP is addressed in a separate question and ask the respondents to re-evaluate this question not taking PEEP, only the positive inspiratory pressure into account.

|  | Strongly disagree | Disagree | No opinion | Agree | Strongly agree |
| --- | --- | --- | --- | --- | --- |
|  |  |  |  |  |  |

Thickening

Does PEEP impact thickening of the diaphragm? q28

Question Round 1: Does PEEP impact thickening of the diaphragm?
Answers Round 1: 64% of respondents (strongly) agreed that PEEP does impact thickening while 36% had no opinion.

Reasons provided were that PEEP changes the initial thickness due to caudal displacement, causing a passive increase in thickness and thus potentially altering the fractional change during a respiratory cycle.

Given this information, we would like the respondents to consider this question again.

|  | Strongly disagree | Disagree | No opinion | Agree | Strongly agree |
| --- | --- | --- | --- | --- | --- |
|  |  |  |  |  |  |

Learning and Expertise

To use diaphragm ultrasound (including diaphragm excursion and thickness) in clinical practice, a minimum of 40 (ideally bilateral) exams, of which at least 20 should be under (indirect) supervision of an experienced teacher. Is this an acceptable guideline ? q29

The number of (supervised) exams necessary to produce reproducible images or reach expertise to guide clinical practice varied strongly, anywhere from 10 to 100. Based on the answers given which were largely centered around 40 exams, we would like to propose a temporary alternative, until evidence on the minimum number is established:

|  | Strongly disagree | Disagree | No opinion | Agree | Strongly agree |
| --- | --- | --- | --- | --- | --- |
|  |  |  |  |  |  |

Daily practice

Is the ability to perform thickness measurements in order to assess thickening a basic ultrasonographic skill that should be required for any intensivist? q30

Question Round 1: Is the ability to perform thickness measurements in order to assess thickening a basic ultrasonographic skill that should be required for any intensivist?

Answers Round 1: 57% of respondents answered that they deem thickness measurements an important skill required for any intensivist, while 28% (strongly-) disagree and 14% no had no opinion.

However, regarding thickening fraction, consensus was reached with 78% that it is an important skill for any intensivist. Given that thickness is part of the thickening assessment, we would like to ask the respondents to reconsider a slightly altered question.

|  | Strongly disagree | Disagree | No opinion | Agree | Strongly agree |
| --- | --- | --- | --- | --- | --- |
|  |  |  |  |  |  |

Thank you, your answers were saved.
