# Supplementary material for: EXpert consensus On Diaphragm UltraSonography in the critically ill (EXODUS): a Delphi consensus statement on the measurement of diaphragm ultrasound-derived parameters in a critical care setting
Source: Crit Care. 2022 Apr 8;26:99. doi: 10.1186/s13054-022-03975-5 (PMC8991486; doi:10.1186/s13054-022-03975-5)

**Diaphragm ultrasonography: Transducer settings and Technique**

*Examples based on the statements in Table 3 of the manuscript*

**Excursion**

Depth:

*“Maximum depth should be adjusted to capture maximum excursion”*

Too little depth: Too much depth:


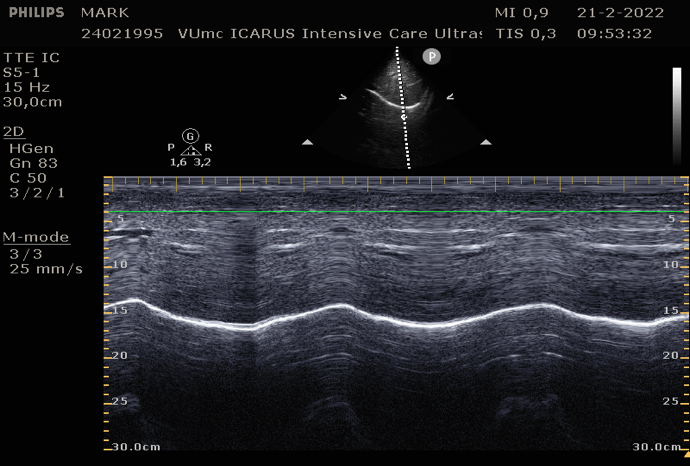

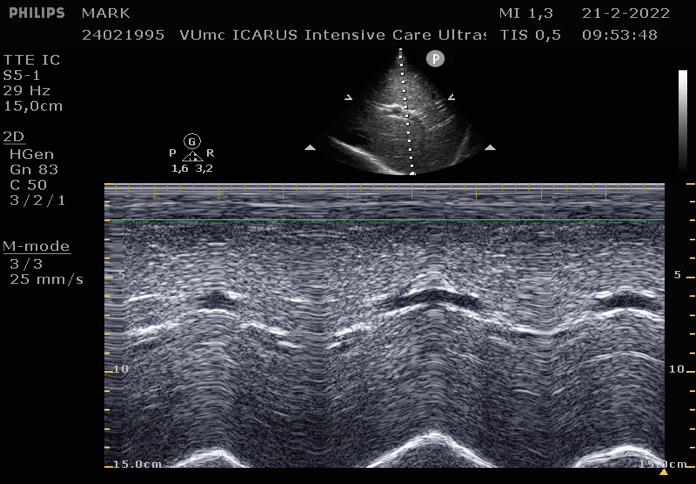


Ideal depth:


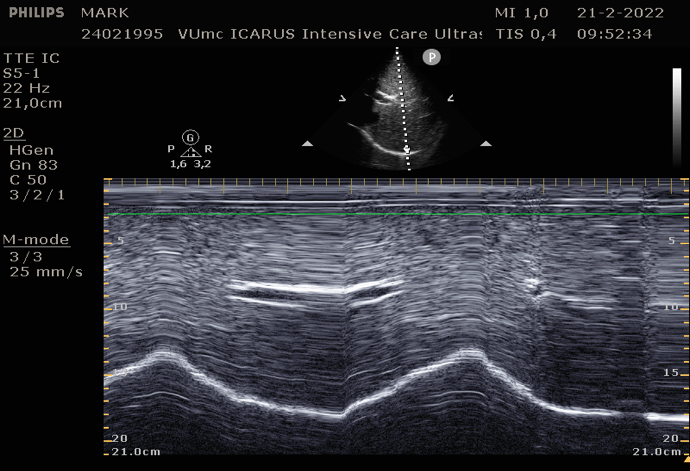


Gain setting:

*“Gain should be adjusted to create ideal contrast with surrounding structures”*

Too little gain: Too much gain:


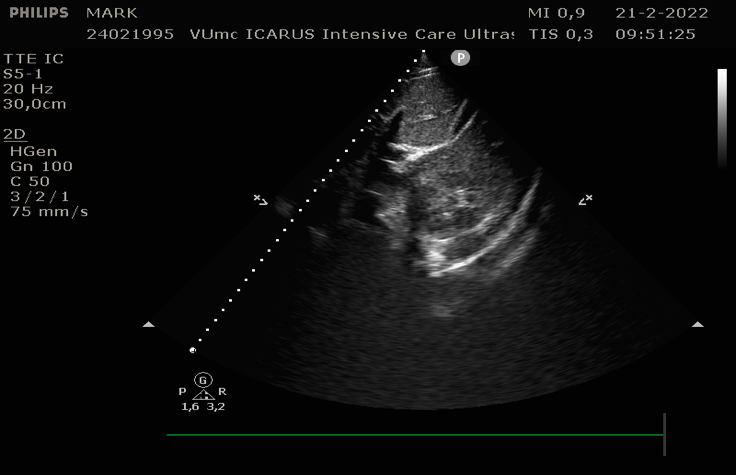

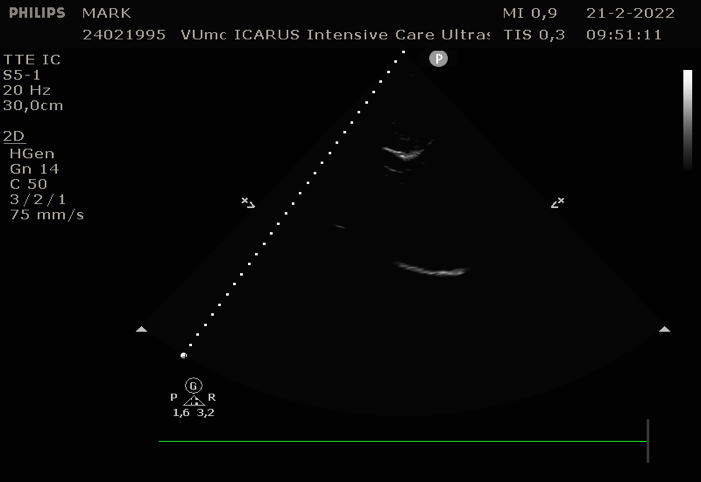


Ideal gain:


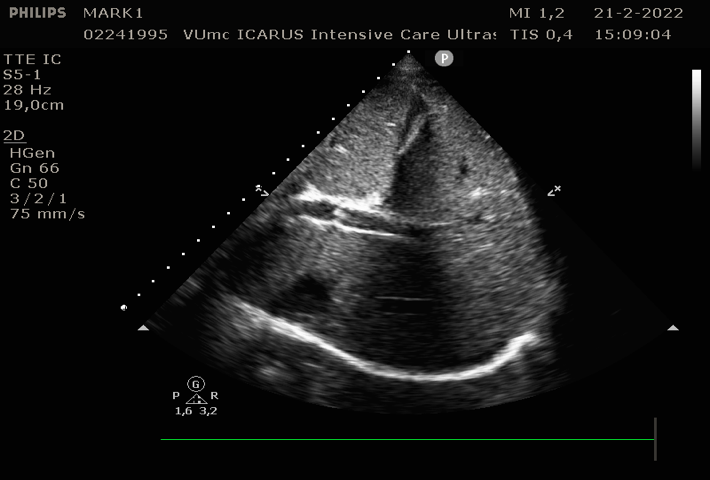


M-Mode vs B-Mode:

*“Measurements are best performed in M-mode and during quiet breathing”*


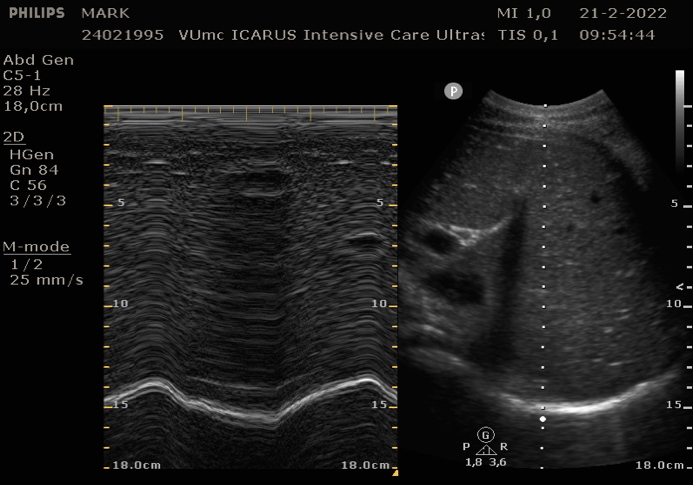


Transducer orientation:

*“The transducer should be aimed at the dome of the diaphragm”*


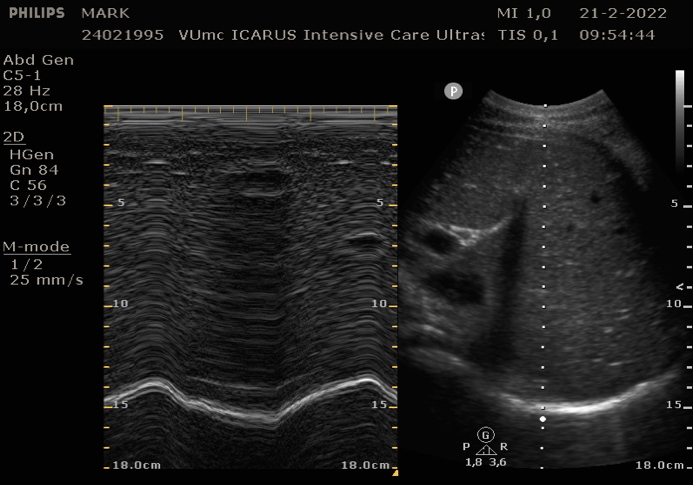


**Thickness**

Transducer placement on the chest wall:

*“The transducer should be placed perpendicular to chest wall, so that all three layers (pleura, peritoneum and fibrous layer) are visible"*

Three layers not visible: Three layers clearly discernable:


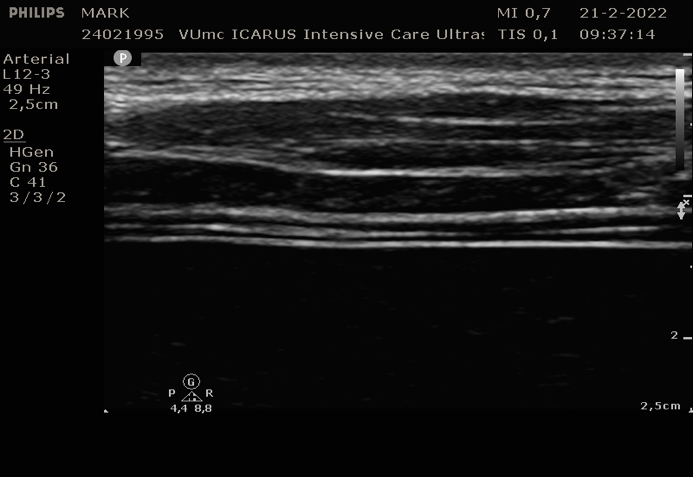

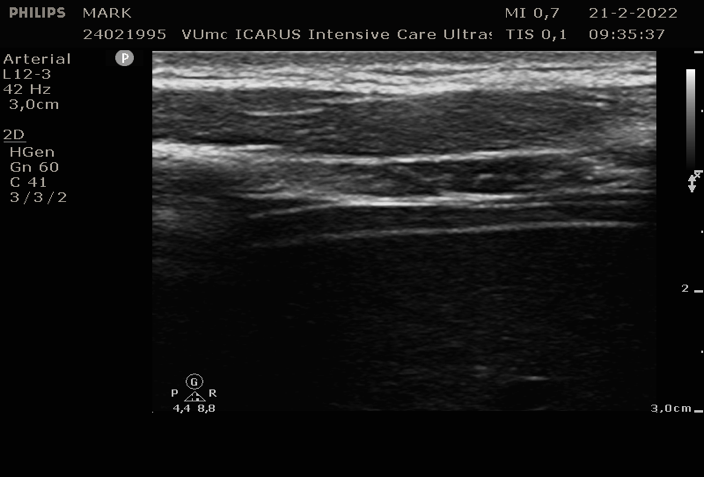


Transducer depth:

*“Depth should be set just below to several centimeters under the diaphragm”*

Too deep: Too shallow:


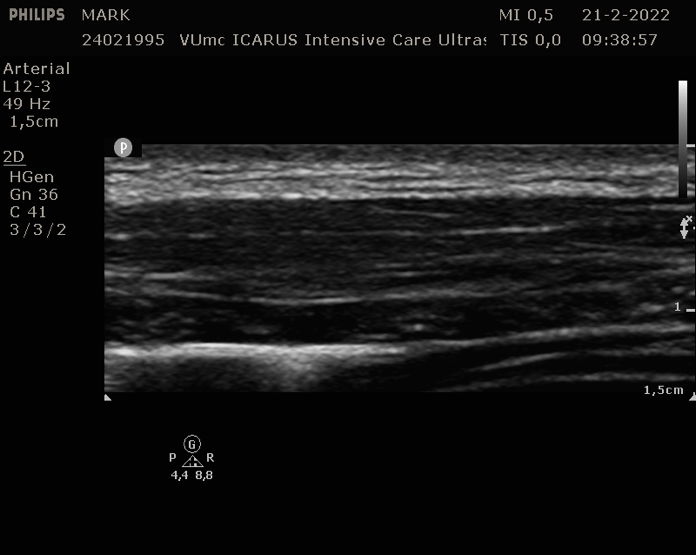


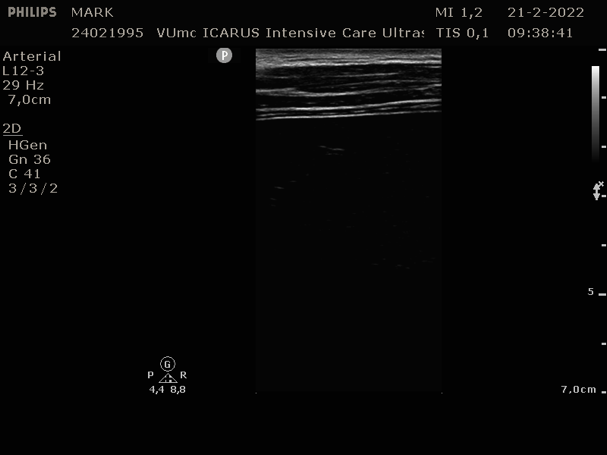


Ideal depth:


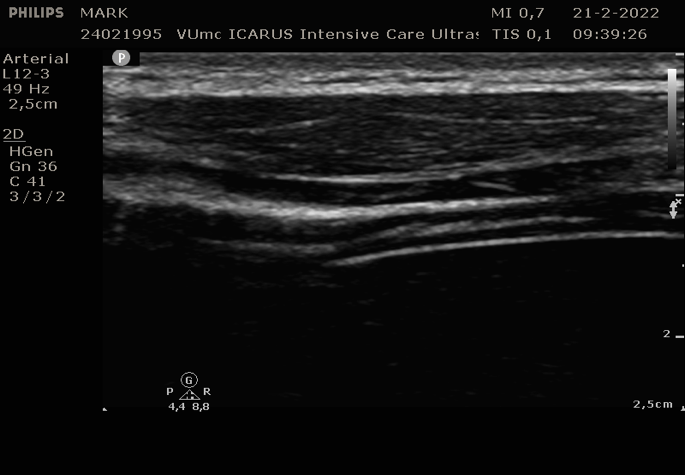


Gain setting:

*“Gain should be adjusted to create ideal contrast with surrounding structures”*

Too little gain: Too much gain:


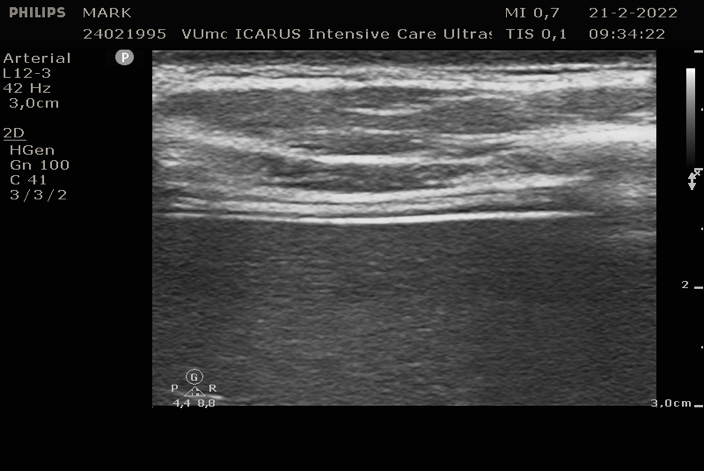

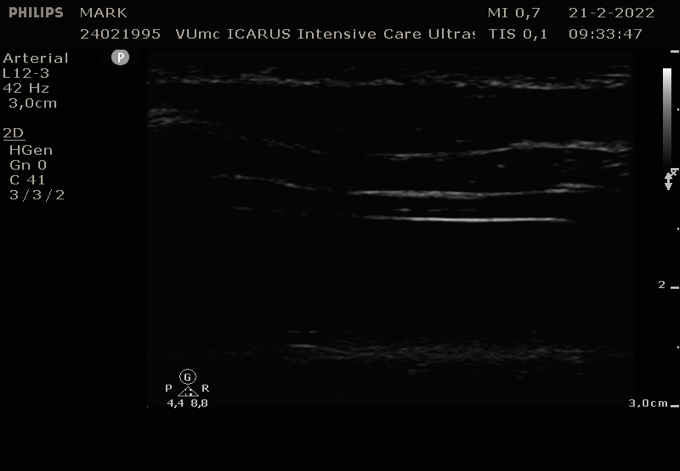


Ideal gain:


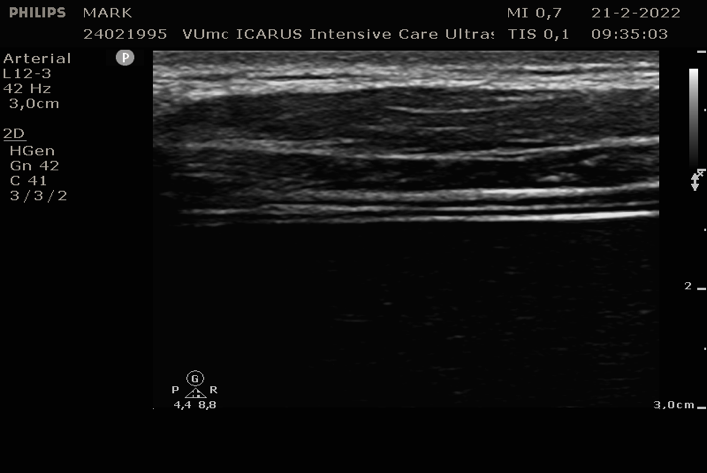


B-mode vs M-mode for thickness measurements:

*“No consensus was achieved for preferring B-mode or M-mode”*


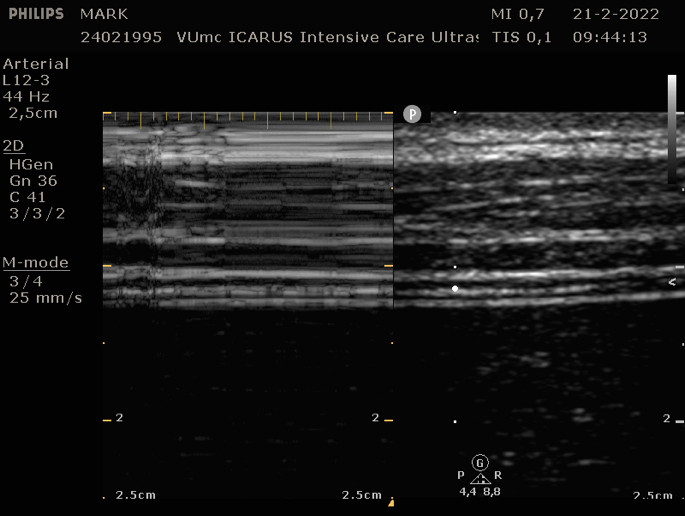


Transducer orientation in regards to intercostal space:

*“No consensus was achieved on transducer orientation to be in line with or perpendicular to the intercostal space”*

In line with intercostal space: Perpendicular to intercostal space:


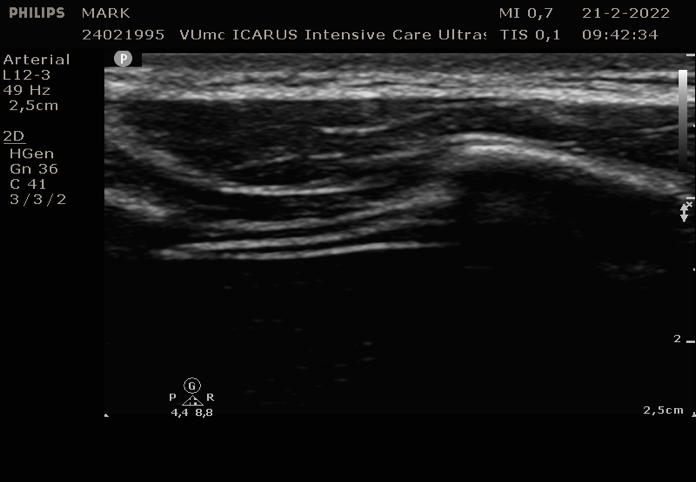

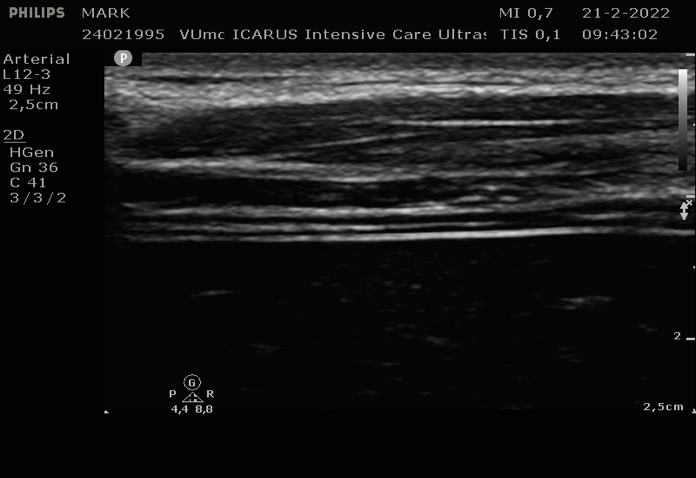


Measuring thickness:

*“Caliper placement should be as close as possible to the pleural and peritoneal line without including these lines in the measurement”*


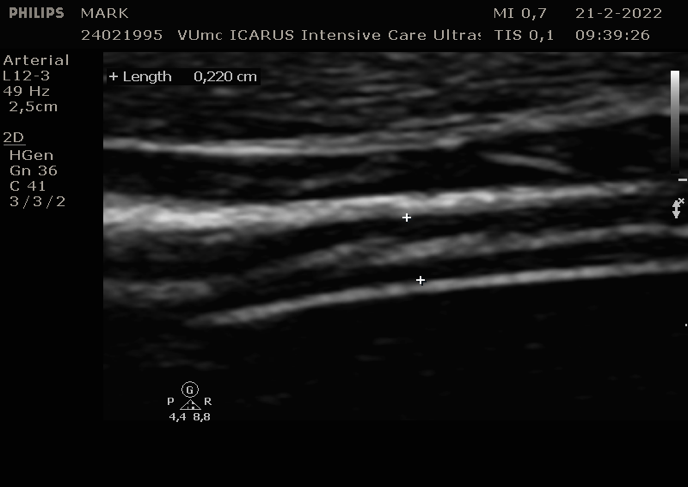

Supplement: Supplementary file 5 — Additional file 5. Visual example of statements. [file 13054_2022_3975_MOESM5_ESM.docx]
